# Synthesis, Characterization, Mössbauer Parameters, and Antitumor Activity of Fe(III) Curcumin Complex

**DOI:** 10.1155/2013/982423

**Published:** 2013-03-27

**Authors:** Mutasim Ibrahim Khalil, Aisha Mohamed Al-Zahem, Maha Hamad Al-Qunaibit

**Affiliations:** Chemistry Department, King Saud University, Riyadh 11451, Saudi Arabia

## Abstract

Curcumin-Fe(III) complex was prepared from Fe(NO_3_)_3_ **·**9H_2_O precursor and curcumin by refluxing a slightly basic methanolic solution of their mixture with the objective of investigating its cytotoxicity. The enol form of curcumin ligand was established by FTIR, UV/Vis, ^1^H NMR, and ^13^C NMR spectroscopy. The as-prepared product was characterized by elemental analysis, FTIR, UV, and Mössbauer spectroscopic techniques. An octahedral high-spin Fe(III) complex was obtained, **δ**, 0.37 mms^−1^; Q.S., 0.79 mms^−1^; no magnetic relaxation was observed at liquid N_2_ temperature, neither reduction of Fe(III). The tested cytotoxicity of the as-prepared complex on four cancer cell lines indicated inhibition of the curcumin activity upon complexing with iron.

## 1. Introduction

The *β* diketone curcumin and curcuminoids (1,7-diaryl-1,6-heptadiene-3,5-diones) which are a group of naturally occurring 1,3-diketones have received considerable attention in the last few decades. This is due to the fact that they possess antitumor [1–3] and antioxidant effects [4] and have a good potential for metal ions complexation. 

It has been reported that metal complexation alters the various physiological properties especially the cytotoxic and antitumor activities of many naturally occurring compounds [5]. It is demonstrated that the coordination of metal ions, for example, Cu(II), Mn(II), Au(III), and so forth, with bioactive ligands can actually improve the pharmaceutical activity of drugs [6, 7].

The Mössbauer metal isotopes, for example, Fe, Au, Ru, Ir, and so forth, form stable complexes with the curcumin ligand [8, 9]. Tonnesen and Greenhill [8] have reported the reduction of Fe(III) to Fe(II) in the presence of curcumin. However, there is no published Mössbauer data on such complexes that could shed light on iron moiety and the correlation between the magnetic, symmetry, and oxidation states of metal ions in such chelates and their biological activity. In this paper we report the Mössbauer data of Fe(curc)_3_ complex, compare it to the well-known Fe(acac)_3_ data, and identify and correlate oxidation state of the central iron metal ion, magnetic relaxation, and structural symmetry of the as-prepared complex to its antitumor cytotoxicity on four cancer cell lines.

## 2. Experimental

### 2.1. Chemicals and Materials

All solvents (Sigma-Aldrich) were reagent grades and were used without further purifications. Curcumin (Sigma-Aldrich and Acros Organics) was extensively analyzed and the Sigma sample form was established. Fe(NO_3_)_3_·9H_2_O (BDH) laboratory reagent was used.

### 2.2. Analytical Instruments

FTIR spectra were recorded in the solid state (KBr pellets) in the range 250–4000 cm^−1^ using a Shimadzu FTIR-8400S, Prestige-21 spectrophotometer. UV/Vis spectra were recorded on a Shimadzu UV-1650 PC spectrophotometer in the range 200–800 nm. ^1^H and ^13^C NMR were recorded on a Jeol Eclipse 400 MHz instrument using TMS external standard (chemical shift *δ* in ppm). C, H, N, O, and iron analysis was carried at Mikroanalytisches Labor Pascher, Remagen Laboratories. The ^57^Fe Mössbauer absorption spectra were recorded at 295 K and 78 K using a Harwell Mössbauer spectrometer. The spectra were refined using least square method.

### 2.3. Cytotoxic Activity Measurements

Cytotoxic activity measurements were carried out in Cairo Cancer Research Center, Egypt. The determination and counting of viable cells were achieved by adding 50 *μ*L of 0.05% trypan blue solution to 50 *μ*L of the single cell suspension. The cells were examined under the inverted microscope using the hemocytometer. Nonstained (viable) cells were counted and the following equation was used to calculate the cell count/mL of cell suspension:

(1) viable cells/mL =number of cells 4 quarters×2(dilution factor)×1044.

The cells were then diluted to give the concentration of single cell suspension required for each experiment.

The percentage of cell survival was calculated as follows:

- survival fraction = O.D. (treated cells)/O.D. (control cells),
- the IC_50_ values are the concentrations of thymoquinone required to produce 50 inhibitions of cell growth. The experiment was repeated 3 times for each cell line.

### 2.4. Synthesis of Fe(III)(acac)_3_ and Fe(III)(curc)_3_ Complexes

**Figure 1:**
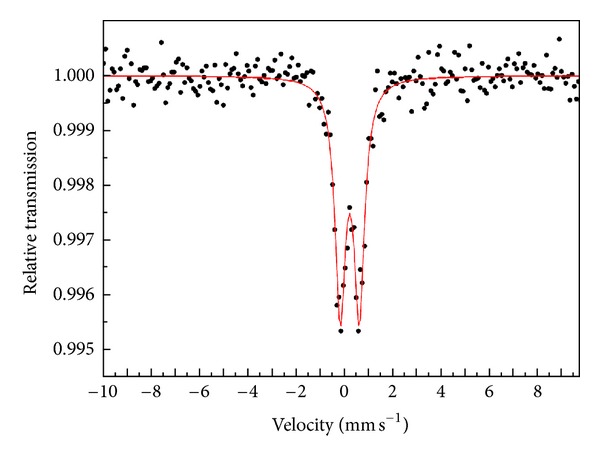
The room Temperature (295 K) Mössbauer spectrum of Fe(III)(curc)_3_.

**Figure 2:**
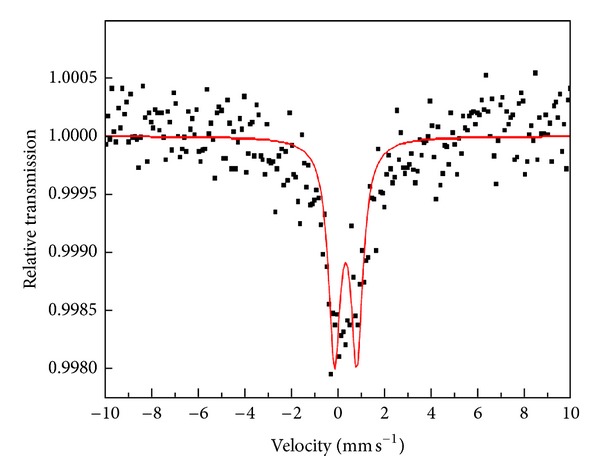
The room temperature (295 K) Mössbauer spectrum of the Fe(III)(acac)_3_.

The Fe(III)(acac)_3_ complex was prepared by dissolving 2.1 g of finely ground iron(III) chloride hexahydrate in 50 mL distilled water in a 250 mL beaker. 0.1 M NaOH solution was added dropwise until the brown precipitate that was formed is completely redissolved. 2.4 mL of acetylacetone in 20 mL ethanol was then added dropwise with continuous magnetic string followed by the addition of 3.3 g sodium acetate in 15 mL distilled water. The whole mixture was heated in a water bath to *≈*80°C and maintained at that temperature for 1 h with rapid stirring. The solution was then cooled to room temperature and cooled further in an ice bath. The red crystals were vacuum filtered and dried in a desiccator [10, 11]. The complex was characterized by elemental analysis, (found; C%, 50.6; H%, 6.3; yield%, 72), FTIR spectroscopy (see supporting information available online at http://dx.doi.org/10.1155/2013/982423), and Mössbauer spectroscopy, Figure 1 and Table 1. Fe(III)(curc)_3_ complex was prepared by refluxing for 3 h in a methanolic mixture solution of 0.6 mmol curcumin and 0.2 mmol Fe(III)(NO_3_)_3_·9H_2_O that was made slightly basic by the addition of few drops of triethylamine. The deep red brown solid product was vacuum filtered, washed with methanol, and dried in vacuum at room temperature overnight. The complex was characterized by elemental analysis, FTIR (supporting information), and Mössbauer spectroscopy, Figure 2 and Table 1. The elemental analytical data are consistent with the formulation: Fe[C_21_H_19_O_6_]_3_·2H_2_O. found, (calc): C%, 63.02, (63.36); H%, 5.06, (5.10); O%, 26.20, (26.79); Fe%, 5.02, (4.68).

## 3. Results and Discussion

The Mössbauer effect is the most reliable technique to identify the oxidation state of iron ions in complexes. The Mössbauer absorption spectrum of the as-prepared curcumin-iron(III) complexes is of similar pattern showing a quadrupole-split doublet of equal intensities and chemical isomer shifts characteristic of octahedral high-spin iron(III) [12–15], Figures 1 and 2 and Table 1.

The absence of any magnetic components at liquid nitrogen temperature rules out the existence of magnetic relaxation. There is no Mössbauer effect evidence that Fe(III) is reduced to Fe(II) in the presence of curcumin as reported in the literature [8]. The Fe^2+^ ion, if present, should show a doublet having an isomer shift of 1.2 mm/s and a quadrupole of 2.9 mm/s [14].

The FTIR and ^1^H NMR spectroscopic measurements have confirmed the enol form of curcumin (supporting information). The FTIR spectrum of Fe(III)(curc)_3 _ complex lost the 3423 cm^−1^ band assigned to *ν*(O–H) of the enol form with the persistence of the phenolic (OH) group vibrational band strongly indicating that it is not coordinated to the metal ion (supporting information). The chemistry of curcumin solution system is pH dependent. Tonnesen and Karlsen [16] have studied the stability of curcumin in the pH range from 1 to 11. They have postulated that curcumin is in equilibrium between three forms, that is, H_3_Curc; H_2_Curc^−^; HCurc^2−^, at pH area 8.2–8.5. They reported a 50% degradation of curcumin in a 0.1% NaOH solution. Pineda et al. [17] measured three acidity constants for curcumin. The one corresponding to the equilibrium H_3_Curc *↔* H_2_Curc^−^ + H^+^ [p*K*_*a*_ = 8.38] was attributed to the acetylacetone-type group. Such a dependence of curcumin system on pH of solution means that the H^+^ ion is involved in reaction mechanism. However, there are some controversies on which proton(s) is involved. While Tonnesen et al. [18] concluded that complex formation between curcumin and iron and the reduction of Fe^3+^ to Fe^2+^ in the presence of curcumin are independent of the phenolic hydroxyl groups in the curcumin molecule, Barclay et al. [19] reported that synthetic nonphenolic curcumin exhibited no antioxidant activity deducing that the H atoms are donated from the phenolic groups. On the other hand, Jovanovic et al. [20] suggested the release of H atoms from CH_2_ group.

**Figure 3:**
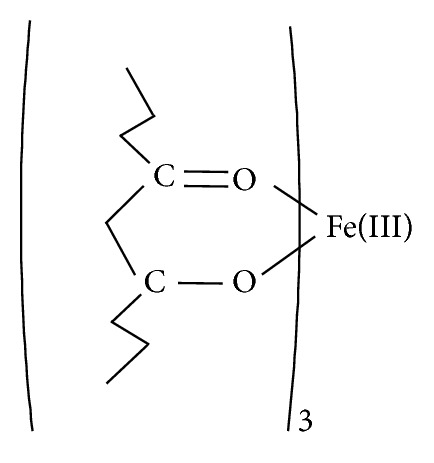
Bidentate coordination of curcumin ligand to Fe(III) ion.

Accordingly, we envisage that the number of protons released by the curcumin enol form determines the type of product. Hence, the three curcumin ligands are coordinated to the iron(III) ion in a bidentate fashion forming the octahedral geometry indicated by the measured Mössbauer parameters, Figure 3.

The antitumor activity of such an established octahedral high-spin iron(III) complex was tested against four cancer cell lines, that is, MCT-7, HepG-2, Hela, and HCT-116. Their activities were compared to doxorubicin (DOX) and pure curcumin. The calculated IC_50_ values indicated that metal complexation inhibits the cytotoxicity of curcumin, Tables 2 and 3.  Table 2 presents the survival fractions of the four cancer cell lines at different concentrations of inhibitors, and the calculated IC_50_ values are presented in Table 3.

Curcumin alone showed higher antioxidant effect towards all four tumor cell lines at IC_50_ values of 3.02 (MCF-7), 3.43 (HepG2), 3.43 (Hela), and 3.81 (HCT-116) *μ*g mL^−1^, than both curcumin-iron(III) complexes (see supporting information). These results are in contrast with the reported concept of synergistic enhancement of ligand effects by combination with ions [5–7, 21–23]. One may relate the lower cytotoxicity of the curcumin-iron complexes compared to curcumin alone to the fact that the central carbon atom with the labile hydrogen is locked and unable to produce oxyradicals unless the curcumin ligand dissociates from the complex. It is more likely that the keto-enol function but not the phenol OH group directs curcumin cytotoxic behavior. 

It is worth mentioning that metal acetates or chlorides are the reagents used by research workers for the synthesis of curcumin-metal complexes although Pineda et al. [17] did not specify the iron salt employed in their study. 

Then, one cannot rule out at this stage the role of the oxidizing nitrate groups in inhibiting the reduction of Fe^3+^ ions by curcumin. It is the reaction suggested by Pineda et al. [17] to take place prior to complex formation. 

The IC_50_ values of both iron complexes, being of comparable values, could be attributed to the antioxidant potential of iron(III) ion. Hence, one can conclude that the diketone system of curcumin appears to be the part of the curcumin molecule involved in the scavenging of oxygen radicals.

## Supplementary Material

Table1 and 2 represent Curcumin 1H and 13C NMR data and assignments, while Figure 1(a,b,c,d) is the FTIR spectra of curcumin and different samples of the curcumin iron complex. Figure 2(a&b) present the 1H & 13C nmr epectra of curcumin. Figure 3 is a diagrametical representation of survival fractions of the different cancer cells versus concentration of inhibitors.

## Figures and Tables

**Table 1:** Mössbauer parameters of Fe(acac)_3_ and Fe(curc)_3_ complexes.

Sample/temperature	I.S. (mm/s)	Q.S. (mm/s)	Γ (mm/s)
Fe(Curcumin)_3_ (295 K)	0.37	0.79	0.51
Fe(Curcumin)_3_ (78 K)	0.46	0.96	0.62
Fe(acac)_3_ (295 K)	0.36	0.82	0.49
Fe(acac)_3_ (78 K)	0.44	0.94	0.33

**Table 2:** Surviving fraction of cancer cells at various concentrations (*µ*g/mL).

Cancer line	Compound	Concentration				
		0	5	12.5	25	50
	Dox	1.000000	0.321921	0.255892	0.170503	0.184712
HCT-116	Curcumin	1.000000	0.337999	0.210925	0.210925	0.298885
	Fe(curc)_3_	1.000000	0.634077	0.596687	0.248749	0.229726

	Dox	1.000000	0.340080	0.301258	0.193985	0.155926
HEPG2	Curcumin	1.000000	0.293962	0.149182	0.236437	0.299768
	Fe(curc)_3_	1.000000	0.969865	0.600591	0.228678	0.312434

	Dox	1.000000	0.203353	0.190643	0.080675	0.096833
Hela	Curcumin	1.000000	0.260466	0.164231	0.186826	0.323229
	Fe(curc)_3_	1.000000	0.933095	0.679533	0.226367	0.363397

	Dox	1.000000	0.194273	0.171715	0.185526	0.201330
MCF7	Curcumin	1.000000	0.140455	0.118242	0.145352	0.250152
	Fe(curc)_3_	1.000000	0.869626	0.737578	0.357802	0.248558

**Table 3:** Cytotoxicity on cancer cell lines.

IC_50_ value (*μ*g/mL)				
Cancer cells				
	MCF-7	HepG-2	Hela	HCT-116

DOX	2.97	4.57	3.64	3.743
Curcumin	3.02	3.43	3.43	3.81
Fe(curc)_3_	20.4	15.8	17.6	16
Fe(acac)_3_	22.0	20.1	21.3	21.0
