# Supplementary material for: Synthesis, Characterization, Mössbauer Parameters, and Antitumor Activity of Fe(III) Curcumin Complex
Source: Bioinorg Chem Appl. 2013 Mar 27;2013:982423. doi: 10.1155/2013/982423 (PMC3623458; doi:10.1155/2013/982423)
Supplement: Supplementary file 1 — Table1 and 2 represent Curcumin 1H and 13C NMR data and assignments, while Figure 1(a,b,c,d) is the FTIR spectra of curcumin and different samples of the curcumin iron complex. Figure 2(a&b) present the 1H & 13C nmr epectra of curcumin. Figure 3 is a diagrametical representation of survival fractions of the different cancer cells versus concentration of inhibitors. [file 982423.f1.doc]

**Supporting Information**

1-Table1. Curcumin ^1^H NMR data and assignments

2-Table2. Curcumin ^13^C NMR data and assignments

3-Figure 1. FTIR spectra: (a) curcumin; (b) first collection of Fe (III) (curc)_3_ ; (c) Second collection; aging collection of Fe (III) (curc)_3_ .

4-Figure 2. (a) & (b) Curcumin ^1^H NMR spectra of curcumin; (c) &(d) ^13^C NMR spectra of curcumin.

5-Figure 3. Diagrametical representation of survival fractions of the different cancer cells versus concentration of inhibitors.

**
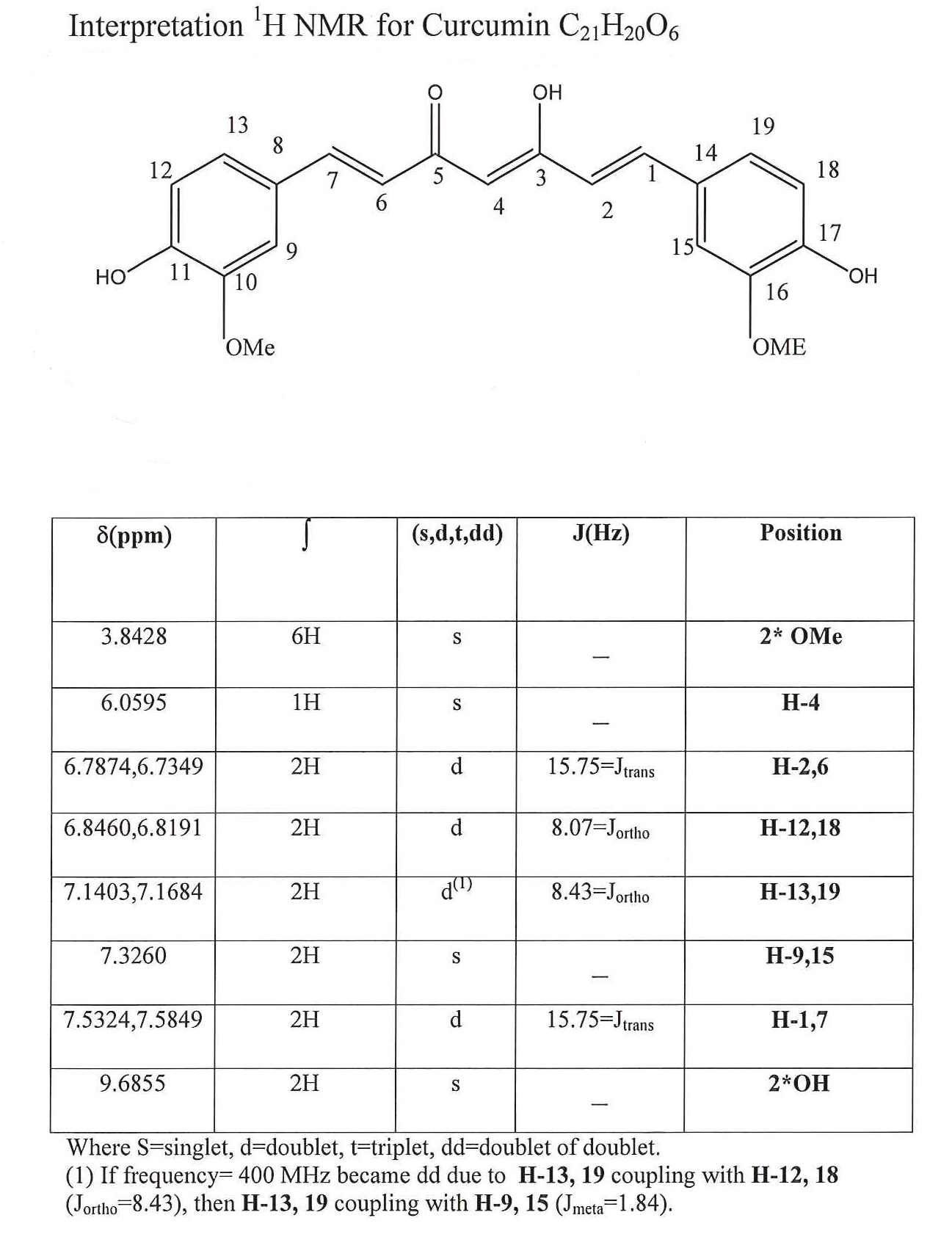
**

**Table 1.**

**
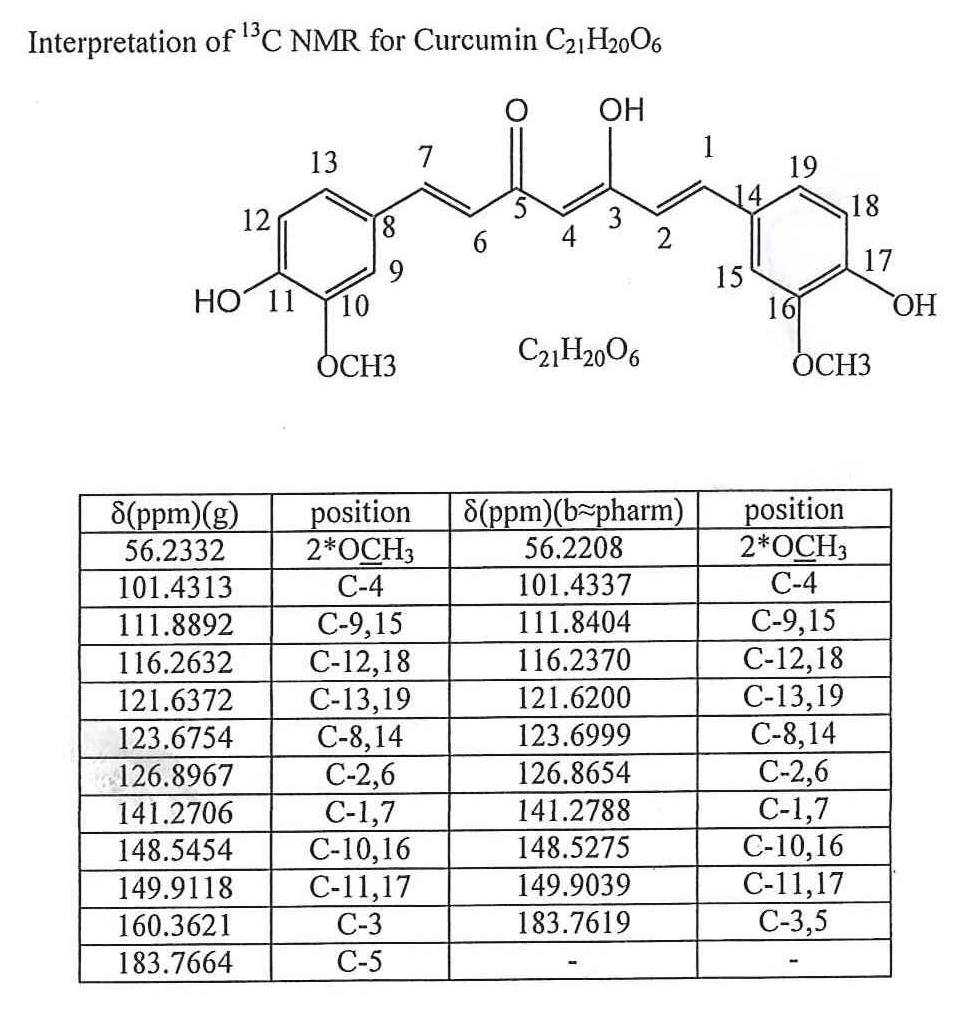
**

**Table 2.**

(a)

(b)

(c)

(d)

  Figure 1.

(a)

(b)

(c)

(d)

Figure 2.

Figure 3.
